# Method for RNA extraction and transcriptomic analysis of single fungal spores

**DOI:** 10.1016/j.mex.2019.12.002

**Published:** 2019-12-04

**Authors:** Ivey A. Geoghegan, Richard D. Emes, David B. Archer, Simon V. Avery

**Affiliations:** a School of Life Sciences, University of Nottingham, University Park, Nottingham NG7 2RD, United Kingdom; b School of Veterinary Medicine and Science, University of Nottingham, Sutton Bonington LE12 5RD, United Kingdom

**Keywords:** RNA extraction, Single-cell transcriptomics, Conidia, Spores, *Aspergillus niger*, Phenotypic heterogeneity

## Abstract

Transcriptomic analysis of single cells has been increasingly in demand in recent years, thanks to technological and methodological advances as well as growing recognition of the importance of individuals in biological systems. However, the majority of these studies have been performed in mammalian cells, due to their ease of lysis and high RNA content. No single cell transcriptomic analysis has yet been described in microbial spores, even though it is known that heterogeneity at the phenotype level exists among individual spores. Transcriptomic analysis of single spores is challenging, in part due to the physically robust nature of the spore wall. This precludes the use of methods commonly used for mammalian cells. Here, we describe a simple method for extraction and amplification of transcripts from single fungal conidia (asexual spores), and its application in single-cell transcriptomics studies. The method can also be used for studies of small numbers of fungal conidia, which may be necessary in the case of limited sample availability, low-abundance transcripts or interest in small subpopulations of conidia.

• The method allows detection of transcripts from single conidia of *Aspergillus niger*
• The method allows detection of genomic DNA from single conidia of *Aspergillus niger*

## Graphical abstract

**Figure:**
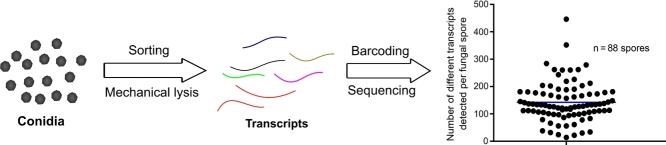

**Specifications Table**

**Table:** 

Subject Area:	Biochemistry, Genetics and Molecular Biology
More specific subject area:	Single-cell transcriptomics
Method name:	RNA extraction and transcriptomic analysis of single fungal spores
Name and reference of original method:	N/A
Resource availability:	**Reagents:**
	Potato Dextrose Agar (PDA)
	Oligo d(T)_20_ primer (Invitrogen Cat no. 18418-020)
	Superscript IV Reverse Transcriptase (Invitrogen Cat no. 18090050)
	RNaseOUT™ Recombinant RNase inhibitor (Invitrogen Cat no. 10777-019)
	Tween 80
	Taq polymerase (New England Bioscience, Cat no. M026S)
	dNTP mix (10 mM each) (New England Bioscience, Cat no. N0447S)
	Nuclease-free water
	BD™ Precise Whole Transcriptome Assay kit
	**Materials:**
	ThermoFisher Savant SPD121P SpeedVac
	ThermoFisher Savant RVT5105 Vapor Trap
	ThermoFisher OFP400 Vacuum pump.
	MP Biomedicals Fastprep-24 homogeniser
	Beckman Coulter MoFlo Astrios cell sorter
	0.5 ml Screw-top microtubes (AlphaLabs CP5913)
	Glass beads (150–212 um) (Sigma G1145)
	Nuclease-free 1.5 ml Eppendorf tubes

## Method details

There is a need to examine the transcriptome at the single spore level. For example, to help explain phenotypes that are heterogeneous among individual spores and which have an important bearing on fungal biology [1,2]. When working with RNA, it is essential to work in an environment that is free from contamination with RNases, to prevent degradation of the sample. This is especially critical when extracting RNA from single cells, due to the minute quantity of RNA present. Throughout this method, observing best-practices for working with RNA [3] will maximize chances of successful RNA recovery from single conidia. Briefly, this should include the use of nuclease-free reagents and pipette tips, working in a clean laboratory environment, keeping samples on ice where possible and using RNase inhibitors. Materials that cannot be purchased as RNase free (for example the glass beads used in this method) can be baked overnight at 180−200 °C to destroy RNases. This method has been optimized using conidia of *A. niger* N402. However, it is likely that this could also be applied to other species of conidia-forming fungi, and potentially adapted for other spore types.

### Harvesting of conidia

1 *A. niger* can be grown either on slopes, or on Petri dishes, either of which will result in plentiful conidia. These can be harvested by adding 5 ml of 0.1 % Tween 80 to the colony and lightly scraping the surface of the colony with a cotton swab (or vortexing in the case of slopes). The presence of Tween 80 helps prevent clumping of conidia.
2 Filter through 40 μm cell strainer, to remove hyphae and larger cell debris.
3 Centrifuge for 10 min at 500 g, discard supernatant and resuspend in 5 ml 0.1 % Tween 80 solution. Spore concentration can be determined by counting on a haemocytometer slide.

### Lysis of conidia and concentration of lysate

Fungal conidia are highly resistant to chemical lysis by buffers that are commonly used for lysis of mammalian cells. Conidia of *A. niger* are also resistant to enzymatic lysis, which prevents the use of protoplasting protocols that have been applied to yeast cells in previous studies [[4], [5], [6]]. This method therefore employs mechanical lysis to release RNA from conidia. However, mechanical lysis is performed in large volumes (250 μl), making it necessary subsequently to concentrate the sample prior to cDNA synthesis and amplification. Concentration of the sample prevents the use of buffer, as the resulting concentrated sample would contain high concentrations of salts and other agents which may inhibit downstream enzymatic processes. For this reason, lysis is performed in water, containing only RNase inhibitor.

1 Sort single conidia into 0.5 ml screw-top microtubes, containing 30−50 mg of glass beads (150–212 μm diameter; other sizes are less effective) and 249 μl of H_2_O + 1 μl (40U) RNaseOUT Recombinant RNase inhibitor. The use of a cell-sorter should guarantee presence of single conidia in the extraction tubes. Alternatively, larger numbers of spores can also be sorted or aliquoted into each tube, if required. If a cell sorter is not available, it is also possible to select single conidia by dilution and microscopic observation. In this case, dilute a suspension of conidia to 1000 conidia/ml and aliquot 1 μl into wells of a flat bottomed 96-well plate. Conidia can be observed using an inverted microscope, and those wells containing single conidia can be recovered with 100 μl H_2_O for transfer to the lysis tube. As successful transfer of the conidium from the well to the lysis tube cannot be guaranteed, it is preferable to use a cell sorter if possible.
2 Lyse in Fastprep for 40 s at 6.5 m/s (other speeds are less effective). Lysis using other methods was not tested although it should be possible to adapt vortexing-based approaches, for example.
3 Centrifuge at 16,000 *g* for 5–10 s.
4 Remove supernatant (∼220 μl) and place in nuclease-free 1.5 ml Eppendorf tube.
5 Place samples in vacuum concentrator, and SpeedVac at ambient temperature to concentrate. It is preferable to concentrate the sample down to a final volume of ∼5 μl, rather than evaporating to complete dryness as resuspension of dried RNA may not be complete.

### cDNA synthesis and amplification

The resulting lysate should be used immediately for cDNA synthesis. Short or long-term storage of conidial lysate is not recommended. This protocol employs Superscript® IV reverse transcriptase, which has been found to be one of the best performing reverse transcriptases for single-cell studies [7].

1 To the 5 μl of cell lysate, add 1 μl of Oligo d(T)_20_ primer, 1 μl of dNTP mix and 6 μl of nuclease-free H_2_O. Mix and briefly centrifuge.
2 In a thermocycler, heat the cell lysate-primer mix at 65 °C for 5 min, then incubate on ice for at least 1 min.
3 Mix the remaining components of the reverse transcriptase reaction in a separate tube (scale appropriately for multiple samples). For each sample: 4 μl of 5 x SSIV buffer, 1 μl of 100 mM DTT, 1 μl of SuperScript® IV reverse transcriptase, 1 μl of nuclease-free H_2_O. Mix and briefly centrifuge.
4 Add the mixed components in step 3 (7 μl) to the cell lysate-primer mix from step 2, and incubate in a thermocycler at 50−55 °C for 10 min, then inactivate the reaction by heating at 80 °C for 10 min.

The entire cDNA reaction (20 μl) should be used in a PCR reaction to detect the transcript of interest. Here, the choice of DNA polymerase is not of paramount importance. This method employs NEB Taq polymerase, but use of alternative DNA polymerases is permissible. Primers should be designed to amplify a region of <200 bp in length, as amplification of longer target sequences may be less efficient. Designing primers to amplify across an intron is also beneficial, as this will allow unintended amplification of genomic DNA to be distinguished from amplification of cDNA (which lacks introns).

1 To the cDNA mixture (20 μl), add primers, dNTPs, buffer, and DNA polymerase to manufacturer’s specifications, to a total reaction volume of 50 μl.
2 Place reaction mixture in a thermocycler, and run according to manufacturer’s instructions. Here, increasing the cycle number (e.g. to 40 cycles) may be necessary to detect transcripts.

## Method validation

### Detection of transcripts in single *A. niger* conidia

**Fig. 1:**
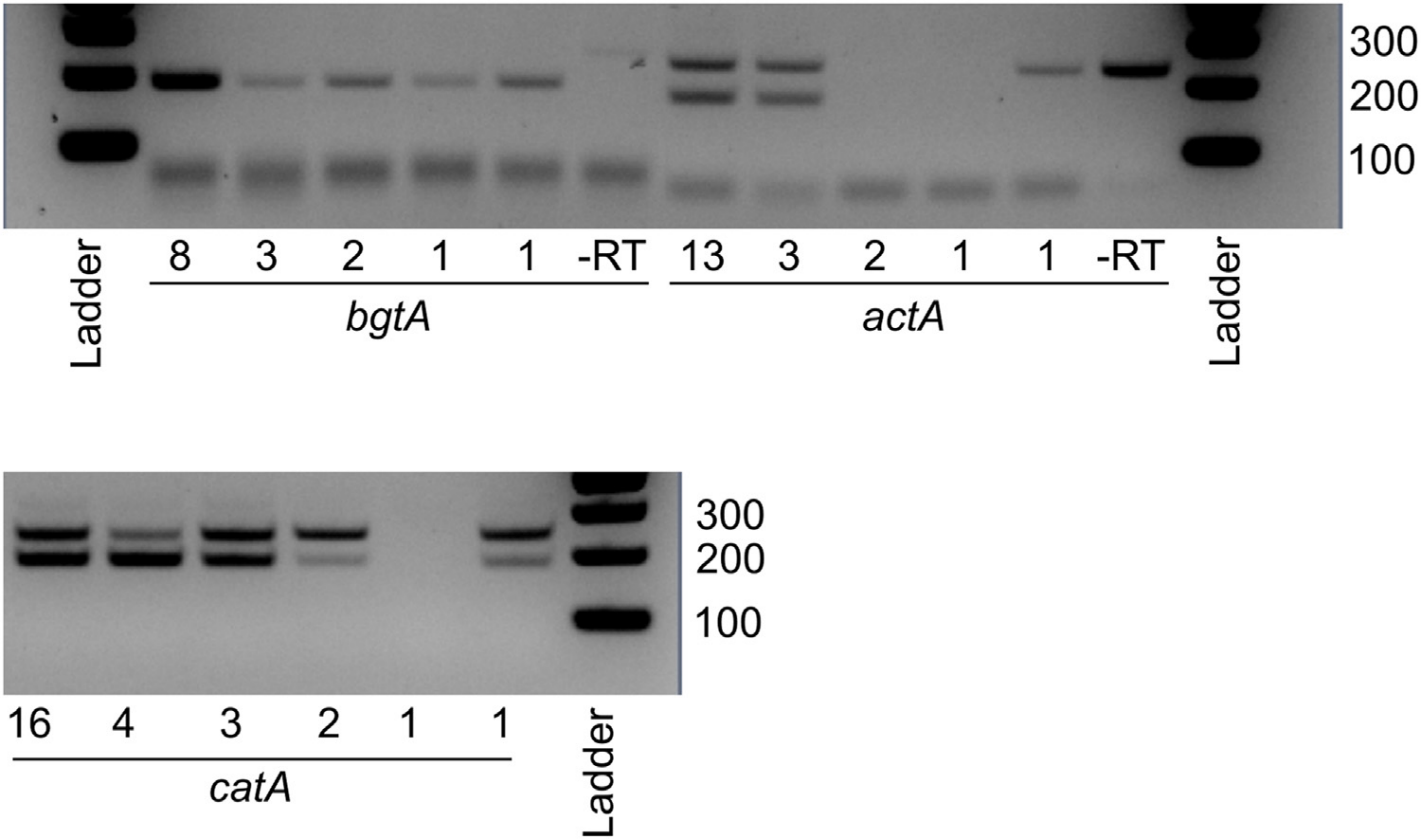
Gel electrophoresis (3 % gel) of amplified cDNA from small numbers of *A. niger* conidia. Three genes were analyzed (*bgtA, actA* and *catA*). The number of conidia extracted is stated below each lane. For the –RT controls, 22 and 26 conidia were extracted for *bgtA* and *actA* respectively. Ladder: 100 bp ladder (New England Bioscience), band sizes indicated at right of pictures. For *actA* and *catA*, the larger-sized fragment corresponds to gDNA. For *bgtA*, a band size of 190 bp is expected for amplification of cDNA, and a band size of 260 bp for amplification of gDNA (faintly visible in the –RT control).

The method described above was used to detect transcripts in small numbers of ungerminated *A. niger* conidia, including single conidia. During the optimization of this method, the small numbers of conidia used were determined by pipetting 1 μl of conidial suspension into a 96-well plate. This allowed counting of conidia in each well using an inverted microscope, and selection of those wells containing the desired number of conidia. Conidia were subsequently transferred to the lysis tubes by washing out the well with 100 μl of nuclease-free water. Fig.1 shows gel electrophoresis of amplified transcripts from three different genes: *bgtA, actA* and *catA.* In the case of *actA* and *catA*, amplification of both genomic DNA and cDNA occurred as evidenced by two bands in each sample. The larger-sized fragment in each case corresponding to gDNA amplification, due to the presence of an intron within the amplicon. In the case of *bgtA*, amplification of cDNA results in a band size of 190 bp, compared with 260 bp when gDNA is amplified. In addition to the size difference between gDNA and cDNA amplification, the inclusion of a minus-RT (reverse transcriptase) control allows simple discrimination between amplification of gDNA or cDNA (the –RT control will not contain cDNA).

As expected, detection of high-abundance transcripts was more successful than detection of low abundance transcripts. Previous transcriptomic analysis of bulk populations of *A. niger* conidia has demonstrated that *bgtA* (encoding 1,3-beta-glucanosyltransferase) and *catA* (catalase A) have high transcript abundance [8]; this is reflected in the detection of these transcripts in single conidia in Fig. 1. Conversely, *actA* (actin) has a lower transcript abundance and transcripts were not successfully detected in samples with either 1 or 2 conidia. Loss of transcript detection can also occur as a result of sample degradation; minute quantities of RNA or DNA are very susceptible to degradation by endogenous or exogenous nucleases.

With low numbers of conidia, transcripts were more commonly detected than gDNA. This may simply reflect the presence of a single copy of the gene of interest in the nucleus of a conidium, whereas transcripts may be present in tens or hundreds of copies in a single conidium. In addition, this method does not make allowances for protecting gDNA from degradation.

### Whole transcriptome analysis of single *A. niger* conidia

The described method was used to attempt a whole transcriptome analysis of single *A. niger* conidia. Numerous different methods have been employed for the analysis of single-cell transcriptomes. However, the mechanical lysis of conidia in the present method is not compatible with microfluidic droplet-based methods as used in Drop-seq, for example [9]. Instead, the mechanical lysis method outlined above is applicable to plate-based single-cell transcriptomics methods (e.g. SCRBseq [10]). In this experiment, we used the BD™ Precise Whole Transcriptome Assay to analyze transcriptomes of single, ungerminated *A. niger* conidia. Lysates of single, ungerminated conidia of *A. niger* were transferred into the wells of the 96-well plate of the BD™ Precise Whole Transcriptome Assay. This assay enables the reverse transcription, barcoding and amplification of whole transcriptomes from single cells, followed by next generation sequencing. The use of Unique Molecular Identifiers (UMIs) enables unique transcripts to be identified and quantified post-amplification, and controls for any amplification bias arising during the amplification steps [11]. Analysis of sequencing data was performed on the Seven Bridges Genomics platform, using the BD™ Precise Whole Transcriptome Assay Analysis pipeline v2.0, comprising FastQC, STAR and HTseq-count, followed by Recursive Substitution Error Correction™ (RSEC) and Distribution-Based Error Correction™ (DBEC) to remove molecular-index errors.

**Fig. 2:**
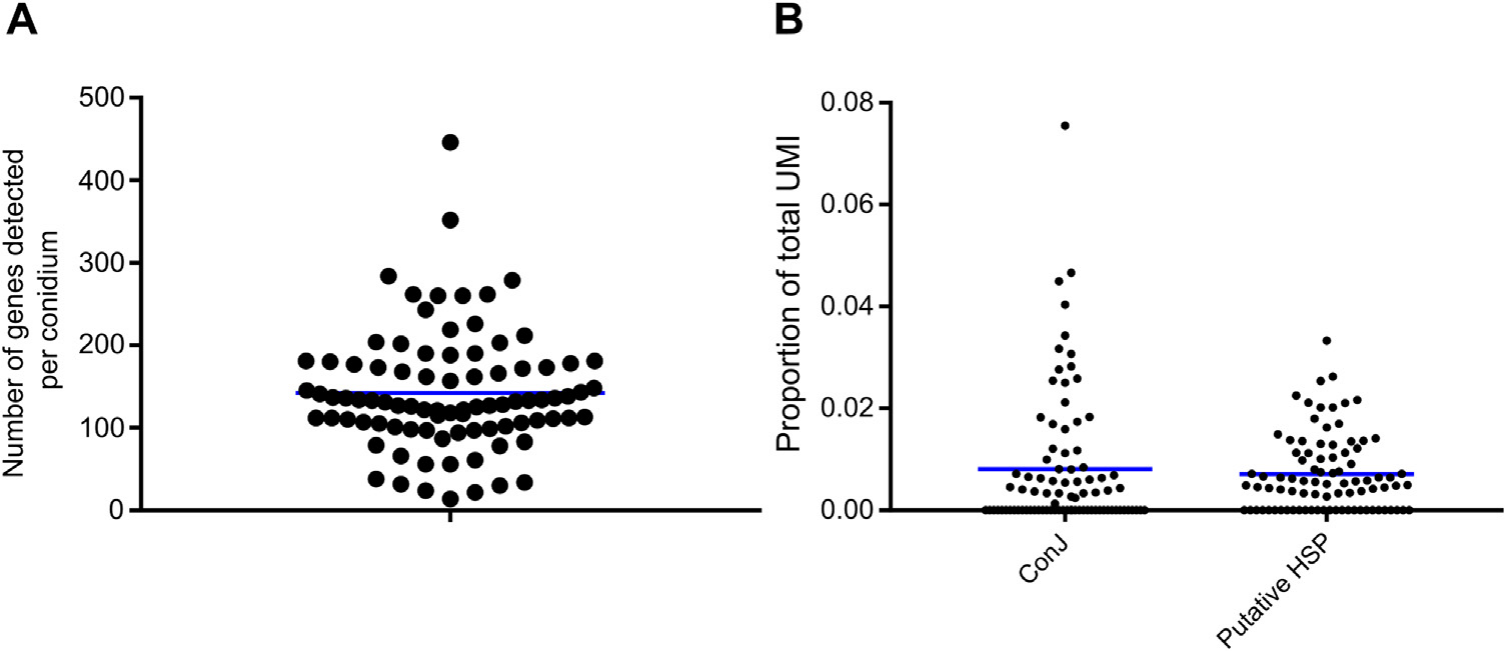
Whole transcriptome analysis of 88 single *A. niger* conidia using BD™ Precise Whole Transcriptome Assay. (A) Number of transcripts (belonging to different genes) detected per conidium (each point represents one conidium). The blue line indicates mean value. (B) Transcript abundance of two genes (ConJ and a putative Heat Shock Protein) in 88 single conidia, expressed as proportion of total Unique Molecular Identifiers (UMIs) detected in each conidium.

We detected transcripts belonging to an average of 140 different genes in each conidium, with the highest number detected 446 and the lowest 14 in particular conidia (Fig. 2A). The transcripts detected were higher abundance transcripts [6]. An example of the transcript abundances for two genes is shown in Fig. 2B. These were detectable in >50 % of the conidia sampled: transcripts of ConJ (An01g10790) were detected in 46 out of 88 conidia, and transcripts of a putative heat shock protein (An06g01610) were detected in 60 out of 88 conidia. These data show that this method allows the transcripts of hundreds of different genes to be detected in single *A. niger* conidia. Previous studies using plate-based methods to study single yeast cells have detected the transcripts of over a thousand different genes [4,5]. However, this may simply reflect greater mRNA abundance/diversity in vegetative cells in those studies compared with dormant spores here; direct comparison with this study is not currently possible as, to our knowledge, this is the first report of single-spore transcriptomics.
